# FAM83B-mediated activation of PI3K/AKT and MAPK signaling cooperates to promote epithelial cell transformation and resistance to targeted therapies

**DOI:** 10.18632/oncotarget.1027

**Published:** 2013-05-11

**Authors:** Rocky Cipriano, Kristy L.S. Miskimen, Benjamin L. Bryson, Chase R. Foy, Courtney A. Bartel, Mark W. Jackson

**Affiliations:** ^1^ Department of Pathology, Case Western Reserve University, Cleveland, Ohio; ^2^ Case Comprehensive Cancer Center, Case Western Reserve University, Cleveland, Ohio

**Keywords:** FAM83B, transformation, drug resistance, CRAF, PI3K

## Abstract

Therapies targeting MAPK and AKT/mTOR signaling are currently being evaluated in clinical trials for several tumor types. However, recent studies suggest that these therapies may be limited due to acquired cancer cell resistance and a small therapeutic index between normal and cancer cells. The identification of novel proteins that are involved in MAPK or AKT/mTOR signaling and differentially expressed between normal and cancer cells will provide mechanistically distinct therapeutic targets with the potential to inhibit these key cancer-associated pathways. We recently identified FAM83B as a novel, previously uncharacterized oncogene capable of hyperactivating MAPK and mTOR signaling and driving the tumorigenicity of immortalized human mammary epithelial cells (HMEC). We show here that elevated FAM83B expression also activates the PI3K/AKT signaling pathway and confers a decreased sensitivity to PI3K, AKT, and mTOR inhibitors. FAM83B co-precipitated with the p85α and p110α subunits of PI3K, as well as AKT, and increased p110α and AKT membrane localization, consistent with elevated PI3K/AKT signaling. In tumor-derived cells harboring elevated FAM83B expression, ablation of FAM83B decreased p110α and AKT membrane localization, suppressed AKT phosphorylation, and diminished proliferation, AIG, and tumorigenicity in vivo. We propose that the level of FAM83B expression may be an important factor to consider when combined therapies targeting MAPK and AKT/mTOR signaling are used. Moreover, the identification of FAM83B as a novel oncogene and its integral involvement in activating PI3K/AKT and MAPK provides a foundation for future therapies aimed at targeting FAM83B in order to suppress the growth of PI3K/AKT- and MAPK-driven cancers.

## INTRODUCTION

The acquisition of persistent growth signaling is a hallmark of cellular transformation that can be conferred by an ever increasing number of mechanisms [1;2]. Often, the aberrant activation of receptor tyrosine kinases (RTK) or downstream effectors such as RAS, MAPK, and PI3K/AKT signaling drive proliferation independently of whether growth factors are present. For example, constitutive RAS activation, conferred either by mutation or upstream RTK hyperactivity, enhances the recruitment of CRAF to the membrane for activation, resulting in MEK1 and MEK2 phosphorylation and subsequently ERK1 and ERK2 phosphorylation [3]. The phosphorylated ERK proteins then activate transcription factors responsible for regulating growth and proliferation. In addition to hyperactivating CRAF, inappropriate RTK or RAS activity also results in increased PI3K-p110 activation, either by IRS (Insulin receptor substrate 1)-mediated p110 recruitment to the receptor or direct binding of a RAS binding domain (RBD) in the p110 catalytic subunit to RAS itself [4].

In numerous breast cancer subtypes, elevated RTK and PI3K signaling is an important driver of transformation [5]. As with MAPK signaling, activation of p110 by mutation or stimulation by RTKs results in a phosphorylation cascade that includes phosphoinositide-dependent protein kinase (PDK-1), AKT, and mammalian target of rapamycin (mTOR). mTOR regulates numerous cell division and growth pathways by modulating transcription, translation and protein degradation [6]. In addition to directly phosphorylating mTOR, AKT also phosphorylates TSC2, which results in a Rheb-mediated interaction between mTOR and Raptor (regulatory associated protein of mTOR) to create mTOR complex 1 (mTORC1). mTORC1 phosphorylates dowstream effectors S6K and 4E-BP1, resulting in increased translation by eIF-4E [7]. Collectively, these processes all promote cell growth and survival in response to RAS-PI3K-AKT activation. Numerous targeted therapies aimed at inhibiting RTKs (HER2, EGFR, MET, KIT, and VEGF) or the downstream effectors described here (RAS, RAF, MEK, PI3K, AKT and mTOR) have been developed and are currently being evaluated in a number of clinical trials [7-9]. However, the complexity of RTK and downstream signaling interactions continues to limit the effectiveness of targeted therapies, since cells can simply reprogram growth and survival signals [10;11]. For these reasons, the identification of novel targets for therapeutic intervention need to be pursued [12].

Recently, we identified FAM83B (Family with Sequence Similarity 83, member B) in a forward genetic screen for genes that drive human mammary epithelial cell (HMEC) transformation [13]. Further, we demonstrated that FAM83B is required for EGFR/RAS-mediated transformation and is a critical component necessary for transmitting signals from activated RAS to downstream MAPK effectors. Mechanistically, the elevated MAPK activation observed in FAM83B-expressing HMEC was due to an increased interaction between FAM83B and CRAF, which dislodged inhibitory 14-3-3 proteins from binding to CRAF and increased CRAF localization at the membrane. Conversely, we found that inhibition of FAM83B from breast cancer cell lines increased CRAF interactions with negative regulatory 14-3-3 proteins, decreased CRAF membrane localization, decreased MEK activity, and decreased tumorigenicity. We show here that elevated FAM83B expression increases PI3K/AKT signaling, which cooperates to transform HMEC and decreases the sensitivity to of HMEC to PI3K, AKT, and mTOR inhibitors. Conversely, ablation of FAM83B from EGFR-dependent breast cancer cells inhibits p110α and AKT membrane localization and AKT phosphorylation. Importantly, the suppression of both the PI3K/AKT and MAPK signaling following FAM83B ablation results in a significant reduction in tumorigenicity, providing critical insight into the potential efficacy of future therapies aimed at targeting FAM83B.

## RESULTS

### FAM83B utilizes multiple RAS effectors to drive HMEC transformation

**Figure 1:**
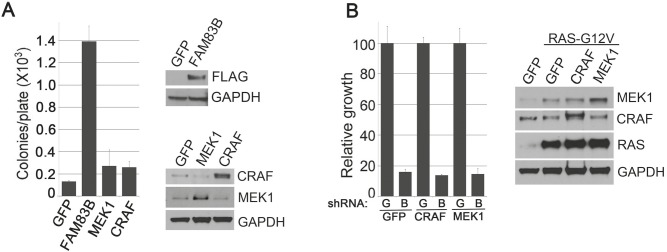
Constitutive CRAF/MEK signaling fails to transform HMEC (a) HME1 cells expressing GFP, FAM83B, constitutively active CRAF, or constitutively active MEK1 were plated in soft agar to assess AIG. Western analysis confirms the expression of CRAF, MEK1, or FAM83B in HME1 cells. (b) HME1 cells expressing RAS were infected with retroviruses encoding cDNAs of *GFP*, constitutively active *CRAF*, or constitutively active *MEK1*. Subsequently, the resulting cell lines were infected with lentiviruses encoding shRNA targeting *GFP* (G) or *FAM83B* (B) and cell growth was assessed 5 days later. Western analysis confirmed the expression of RAS, CRAF, and MEK1 in HME1 cells.

We recently identified FAM83B in a Validation-Based Insertional Mutagenesis (VBIM) screen based on its ability to promote the anchorage-independent growth (AIG) and tumorigenicity of immortalized HMEC in a manner similar to RAS-G12V [13]. Subsequent analysis defined FAM83B as a critical intermediary in CRAF/MAPK signaling. To determine whether FAM83B-mediated CRAF activation is sufficient to drive HMEC transformation or whether FAM83B was activating additional signaling, we examined whether constitutively active CRAF (CA-CRAF) or MEK1 (CA-MEK1) proteins alone could drive HME1 transformation. Retroviruses encoding CA-CRAF, CA-MEK1, or FAM83B (as a positive control) were used to infect HME1 cells. Western analysis was used to confirm the appropriate expression of each protein, and each HME1 derivative was examined for AIG (Fig. 1A). As expected, FAM83B expression resulted in efficient AIG; however, neither CA-CRAF nor CA-MEK1 alone was sufficient to promote the AIG of HME1 cells.

Our previous studies demonstrated that RAS-G12V-transformed HME1 cells require sustained FAM83B expression to maintain a transformed phenotype, implicating FAM83B as a key component in RAS/MAPK signaling. We hypothesized that, if FAM83B functions solely as an activator of MAPK signaling, then CA-CRAF or CA-MEK1 should functionally compensate for FAM83B and allow sustained proliferation following shRNA-mediated FAM83B ablation. To test this hypothesis, RAS-G12V-transformed HME1 cells were infected with retroviruses encoding GFP, CA-CRAF, or CA-MEK1, then subsequently infected with lentiviruses encoding shRNAs targeting *GFP* (G) or *FAM83B* (B) before assessing cell number. Western analysis confirmed the expression of CA-CRAF and CA-MEK1 in the RAS-G12V-transformed HME1 cells. As expected, ablation of FAM83B resulted in a significant reduction in cell number (Fig. 1B). Importantly, neither constitutively active CRAF nor constitutively active MEK1 was able to rescue RAS-expressing cells from the growth inhibition mediated by FAM83B ablation (Fig. 1B). These results demonstrate that constitutive CRAF or MEK are not sufficient to drive HME1 transformation alone, and cannot compensate for the loss of FAM83B, arguing that elevated FAM83B expression modulates additional effectors necessary to drive transformation.

To further define the signaling pathways that contribute to HME1 transformation, we next compared FAM83B to RAS-G12V or various RAS-G12V point mutants capable of activating specific effector pathways, including RAF (T35S), PI3K (Y40C), or RAL-GEF (E37G). Western analysis confirmed the expression of the RAS-V12G proteins (Supplementary Fig. 1A). Both FAM83B and RAS-G12V expression resulted in efficient AIG, while each of the RAS-G12V mutants capable of specific effector pathway activation failed to produce significant AIG, further supporting the conclusion that HME1 transformation requires multiple effector pathways (Supplementary Fig. 1B). In addition, FAM83B-expressing HME1 cells possess the ability to grow in the absence of Mammary Epithelial Growth Supplement (MEGs), which mimics a low growth factor environment *in vivo* (Supplementary Fig. 1B). This phenotype could not be recapitulated by HMEC expressing RAS-G12V mutants capable of activating single effector pathways (Supplementary Fig. 1B). Taken together, our results suggest that FAM83B has additional, growth promoting and transforming functions that are independent of the previously described RAF/MAPK activation.

In addition to activating CRAF signaling, FAM83B expression results in elevated conventional phospholipase D (PLD) activity. Inhibition of either CRAF or PLD1 suppresses FAM83B-mediated transformation [13]. Therefore, we hypothesized that the combination of constitutive CRAF activity and elevated PLD activity would recapitulate FAM83B-mediated transformation. To test this hypothesis, HME1 cells expressing GFP or constitutively active CRAF were infected with retroviruses encoding GFP, PLD1, or PLD2. Western analysis confirmed the expression of each protein and AIG was assessed (Supplementary Fig. 2A). Again, expression of FAM83B promoted efficient AIG while expression of PLD1 or PLD2 alone or in combination with constitutively active CRAF was insufficient to promote AIG. Similarly, only FAM83B expression conferred sustained proliferation in the absence of Mammary Epithelial Growth Supplement (MEGs) (Supplementary Fig. 2B). Thus, while CRAF and PLD activity are required for FAM83B-mediated transformation, simply co-activating the two pathways is insufficient to recapitulate the FAM83B phenotypes.

### Defining the signaling required to recapitulate FAM83B phenotypes

**Figure 2:**
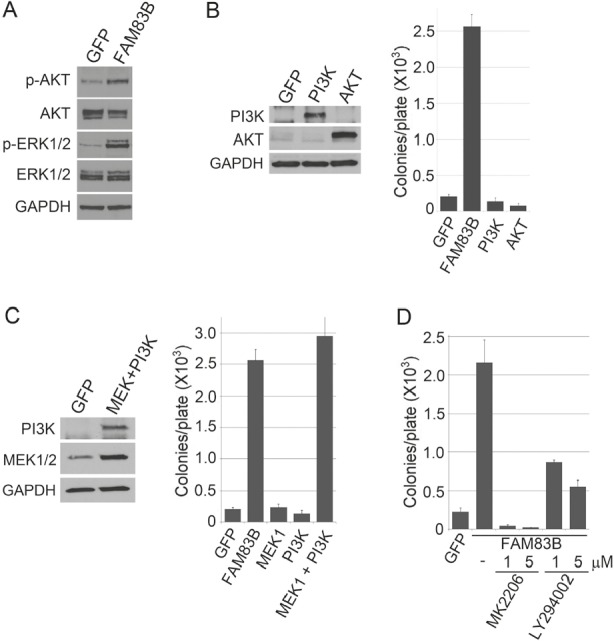
FAM83B requires PI3K/AKT and CRAF/MEK activity to induce AIG (a) Western analysis of FAM83B or GFP expressing HME1 cells for p-AKT (S473), p-ERK1/2 (Thr 202/Tyr 204), ERK1/2, AKT, and GAPDH. (b) HME1 cells expressing GFP, FAM83B, constitutively active AKT, or constitutively active PI3K were assessed for AIG and western analysis. (c) HME1 cells expressing GFP, FAM83B, constitutively active MEK1, constitutively active PI3K, or constitutively active MEK1 and constitutively active PI3K together were assessed for AIG and western analysis. (d) AKT/PI3K inhibition abolishes FAM83B-mediated AIG. HME1 cells expressing GFP or FAM83B were analyzed for AIG in the presence of a PI3K inhibitor (LY294002) or an AKT inhibitor (MK2206).

FAM83B is one of eight members of a protein family (FAM83), based solely on the presence of an N-terminal Domain of Unknown Function (DUF1669). A recent report by Lee *et. al* demonstrated that another member of this family, FAM83A, can activate the PI3K signaling axis, leading to AKT phosphorylation [14]. To determine whether FAM83B expression could promote AKT phosphorylation, FAM83B-expressing HME1 cells were examined using Western analysis to detect AKT phosphorylated on Serine 473 (Fig. 2A). Importantly, AKT phosphorylation was elevated in FAM83B-expressing HME1 cells, similar to ERK1/2 as we previously reported. To examine whether elevated signaling through the PI3K signaling axis is sufficient to drive HME1 cells to grow anchorage independently, HME1 cells expressing GFP, FAM83B, constitutively active AKT (CA-AKT), or constitutively active PI3K (CA-PI3K) were created. Expression of CA-AKT and CA-PI3K were confirmed by Western analysis, and AIG was assessed (Fig. 2B). As observed with CA-RAF and CA-MEK, cells expressing CA-PI3K alone or CA-AKT alone were unable to grow anchorage-independently, arguing that simply elevating AKT activity is insufficient for transformation. Next, we examined whether activation of CRAF/MAPK and PI3K/AKT signaling together would recapitulate the function of FAM83B to drive a transformed phenotype. HME1 cells expressing GFP, FAM83B, CA-MEK1 alone, CA-PI3K alone, or CA-MEK1 and CA-PI3K together were created, analyzed by Western analysis and assessed for AIG (Fig. 2C). Importantly, combined activation of the CRAF/MAPK and PI3K/AKT pathway together resulted in AIG similar to FAM83B expression.

**Figure 3:**
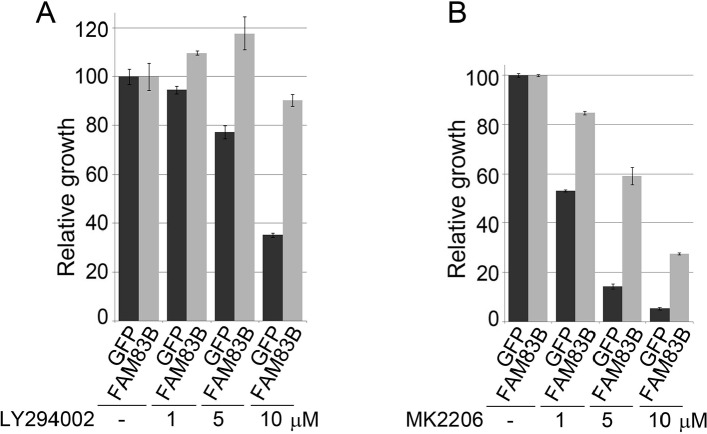
FAM83B expression confers resistance to therapeutic inhibition of the PI3K/AKT signaling pathway (a) HME1 cells expressing GFP or FAM83B were plated in the presence and absence of a PI3K inhibitor LY294002 (1, 5, and 10 μM) and cell number quantified 5 days later. (b) HME1 cells expressing GFP or FAM83B were plated in the presence and absence of an AKT inhibitor MK2206 (1, 5, and 10 μM) and cell number quantified 5 days later.

Having defined PI3K/AKT signaling as a key effector pathway that cooperates with MAPK signaling to transform HME1 cells, we next sought to confirm that FAM83B-mediated transformation requires sustained PI3K/AKT activity. HME1 cells expressing GFP or FAM83B were analyzed for AIG in the presence of a PI3K inhibitor (LY294002) or an AKT inhibitor (MK2206) (Fig. 2D). FAM83B-mediated AIG was significantly diminished following inhibition of either PI3K or AKT activity, confirming that the hyperactivated AKT observed in FAM83B-expressing cells is critical for FAM83B-mediated transformation. Furthermore, elevated FAM83B expression diminished the sensitivity of HME1 cells to therapeutic inhibition of the PI3K/AKT signaling axis, either by LY294002 (1, 5, and 10 μM) or MK2206 (1, 5, and 10 μM), with as much as a 4-fold increase in cell number when compared to control GFP-expressing cells (Fig. 3A and 3B). In addition, we previously demonstrated that the phosphorylation of mTOR effectors S6K and 4E-BP1 was increased in cells harboring elevated FAM83B expression. The hyperactivation of PI3K/AKT by elevated FAM83B expression provides an explanation for the increased phosphorylation of these mTOR effectors [13]. Taken together with our previous findings that elevated FAM83B expression also decreased the sensitivity to mTOR inhibition, CRAF inhibition, and MEK inhibition, we conclude that FAM83B-mediated AKT and MAPK hyperactivation renders transformed cells less sensitive to therapies targeting each pathway [13].

### FAM83B inhibition suppresses cancer cell growth and decreases AKT phosphoryation by altering the subcellular location of multiple PI3K signaling components

**Figure 4:**
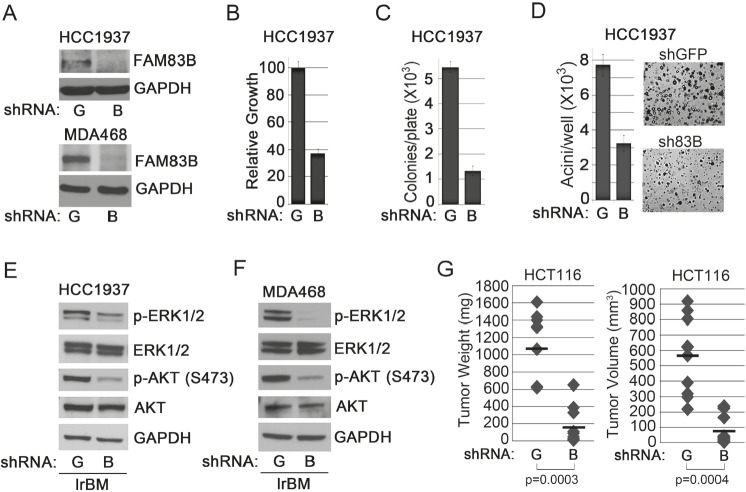
FAM83B inhibition suppresses EGFR- and RAS-driven cancer cell growth (a) Western analysis of HCC1937 and MDA468 cells expressing shRNAs targeting GFP (G) or FAM83B (B) for FAM83B and GAPDH. (b) HCC1937 cells expressing shRNAs targeting GFP (G) or FAM83B (B) were plated and cell number quantified 5 days later. (c and d) HCC1937 cells expressing shRNAs targeting GFP (G) or FAM83B (B) were assessed for AIG or were grown as 3-dimensional (3D) cultures in laminin-rich basement membrane (lrBM) for 10 days. Pictures were taken and acini number determined using MetaMorph image quantitation software. (e and f) HCC1937 and MDA468 cells expressing shRNAs targeting GFP (G) or FAM83B (B) were grown in lrBM for 10 days and western analysis was performed for p-AKT (S473), p-ERK1/2 (Thr 202/Tyr 204), ERK1/2, AKT, and GAPDH. (g) HCT116 cells, which harbor a KRAS-G13D mutation, were infected with shRNA targeting GFP or FAM83B and injected subcutaneously into immunocompromised mice to assess tumor formation.

ShRNA-mediated FAM83B ablation inhibits MAPK signaling and suppresses the growth of tumor-derived breast cancer cells harboring elevated EGFR signaling or mutant RAS [13]. To determine whether PI3K/AKT signaling is also inhibited in cancer cells following FAM83B ablation, shRNA targeting *FAM83B* (B) or *GFP* (G) was delivered to HCC1937 and MDA468 breast cancer cells (harboring elevated EGFR). The efficiency of FAM83B knock-down was examined by Western analysis (Fig. 4A) and the proliferation, AIG, and acinar growth in laminin-rich basement membrane (lrBM) was assessed. The growth of both HCC1937 and MDA468 cells was suppressed in all three assays following FAM83B inhibition (Fig. 4B-D; [13]). Moreover, ablation of FAM83B resulted in a strong suppression of both AKT and ERK phosphorylation (Fig. 4E and 4F). Together, our data demonstrates that targeted suppression of FAM83B can simultaneously inhibit two key growth regulatory pathways downstream of EGFR, namely PI3K/AKT and MAPK. In addition, we previously noted that HME1 cells expressing mutant RAS were sensitive to FAM83B ablation, implicating FAM83B as a key signaling intermediary downstream of mutant RAS. To confirm the requirement for FAM83B in mutant RAS-mediated tumorigenicity *in vivo*, we infected HCT116 colorectal cancer cells (KRAS-G13D and elevated FAM83B mRNA) with lentiviruses expressing shRNAs targeting *FAM83B* (B) or *GFP* (G), injected NOD/SCID mice, and measured tumor formation after two weeks. Again, suppression of FAM83B resulted in a significant inhibition of both tumor volume and tumor weight when compared to control, shGFP-expressing cells (Fig. 4G). Taken together, our studies define both PI3K/AKT and MAPK signaling as key effectors induced by elevated FAM83B expression and required for efficient FAM83B- and EGFR/RAS-mediated transformation.

**Figure 5:**
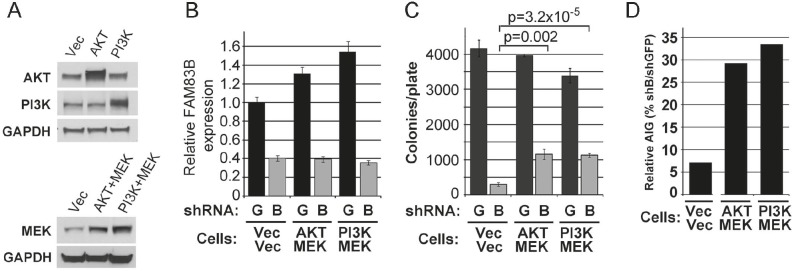
Constitutively active PI3K/AKT and MEK signaling partially rescue MDA468 cells from growth suppression following FAM83B ablation MDA468 cells expressing constitutively active AKT or constitutively active PI3K with constitutively active MEK1 were created. (a) Western analysis of the CA-AKT/CA-MEK- and CA-PI3K/CA-MEK-expressing MDA468 cells. (b-d) Each MDA468 derivative was infected with lentiviruses encoding shRNAs targeting GFP or FAM83B, and analyzed for FAM83B expression by real-time PCR (c) prior to plating into soft agar to assess AIG (c). Relative AIG (defined as the percentage of shFAM83B growth relative to the shGFP control) between the MDA468 derivatives was plotted (d).

We next performed a genetic experiment to determine whether constitutively active AKT (CA-AKT), or constitutively active PI3K (CA-PI3K), when combined with constitutively active MEK1 (CA-MEK) could prevent the growth suppression observed following FAM83B ablation. For this, MDA468 cells expressing CA-AKT and CA-MEK or CA-PI3K and CA-MEK were created, infected with lentiviruses encoding FAM83B-shRNA, and AIG was measured (Fig. 5). While shRNA-mediated FAM83B ablation was equivalent among the MDA468 derivatives (Fig. 5B), the presence of CA-AKT/CA-MEK or CA-PI3K/CA-MEK resulted in a significant increase in AIG when compared to control cells (p=0.0024 and p=0.000032, respectively), although this was not a complete rescue (Fig. 5C and 5D).

**Figure 6:**
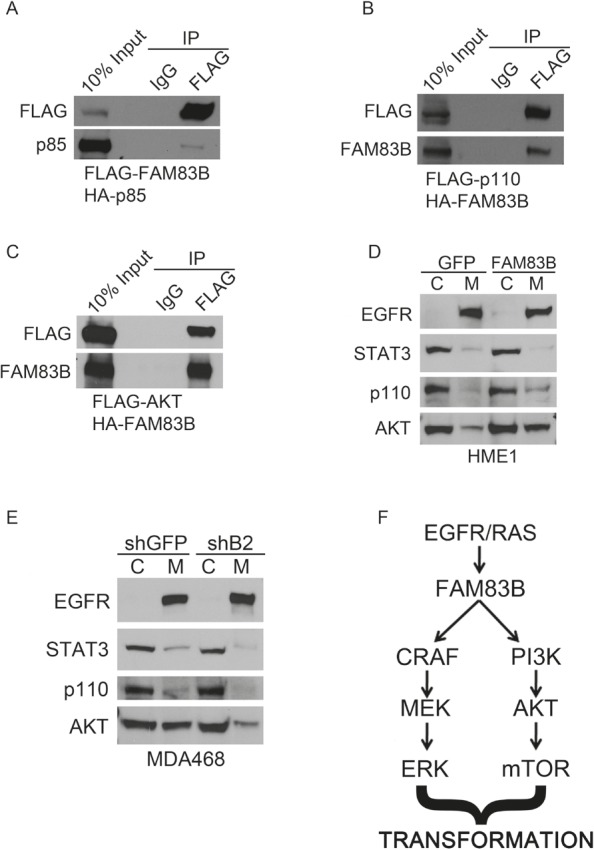
FAM83B expression alters the subcellular location of multiple PI3K signaling components (a) 293T cells were transfected with an expression constructs encoding HA-p85 and FLAG-FAM83B as indicated. Immunoprecipitation was performed using a FLAG antibody, and precipitated proteins analyzed by Western analysis to determine the amount of p85 bound to FAM83B. (b) 293T cells were transfected with an expression constructs encoding FLAG-p110 and HA-FAM83B as indicated. Immunoprecipitation was performed using a FLAG antibody, and precipitated proteins analyzed by Western analysis to determine the amount of FAM83B bound to p110. (c) 293T cells were transfected with an expression constructs encoding FLAG-AKT and HA-FAM83B as indicated. Immunoprecipitation was performed using a FLAG antibody, and precipitated proteins analyzed by Western analysis to determine the amount of FAM83B bound to AKT. (d) A subcellular protein fractionation kit (Thermo Scientific) was used to isolate cytoplasmic (C) and membrane (M) fractions from HME1 cells expressing GFP or FAM83B. Normalized portions of each extract were analyzed by Western blotting using antibodies against EGFR, STAT3, p110 and AKT. (e) A subcellular protein fractionation kit (Thermo Scientific) was used to isolate cytoplasmic (C) and membrane (M) fractions from MDA468 cells expressing shRNAS targeting GFP or FAM83B (B2). Normalized portions of each extract were analyzed by Western blotting using antibodies against EGFR, STAT3, p110 and AKT. (f) A model of FAM83B-mediated transformation.

To define the mechanism by which FAM83B activates PI3K/AKT signaling, we examined whether PI3K signaling components co-precipitated with FAM83B. We first examined whether FAM83B co-precipitated PI3K subunits by co-expressing FAM83B together with either the p85α regulatory subunit of PI3K or the p110α catalytic subunit. FAM83B was able to co-precipitate with both the p85α and p110α subunits (Fig. 6A and 6B). Using a similar assay, we determined that AKT was also able to co-precipitate with FAM83B (Fig. 6C). In addition, we observed a significant increase in the amount of membrane-localized p110α and AKT in FAM83B-expressing cells compared to control cells (Fig. 6D). Conversely, ablation of FAM83B expression from MDA468 cells, which suppressed the growth and tumorigenicity of this cell line, resulted in a decrease in p110α and AKT membrane localization and subsequently a decrease in AKT signaling (Fig. 6E and 4G). Together, our data suggest that the increased level of FAM83B observed in many cancers results in increased localization of p110α to the membrane, and activation of downstream AKT/mTOR signaling (Fig 6D and 2A) and suggests that the therapeutic targeting of FAM83B may suppress PI3K/AKT activation and ultimately inhibit tumor growth.

## DISCUSSION

Understanding the mechanism by which elevated FAM83B expression drives cellular transformation will provide the foundation for future therapies aimed at suppressing the key functions of this novel oncogene. Previously, we demonstrated that FAM83B expression elevates MAPK activity by increasing CRAF membrane localization. Based on our observations, we put forth a hypothesis that FAM83B was a key regulator of RAF/MAPK signaling, and that its function was simply to connect upstream EGFR/RAS with RAS/MAPK activation. Surprisingly, when we began examining the requirements for HME1 transformation, we noted that the expression of activated CRAF or MEK1 failed to induce AIG comparable to FAM83B. Additionally, the expression of RAS-G12V mutants only capable of activating a single downstream effector pathway (RAF, PI3K, and RAL) in HME1 cells failed to induce AIG comparable to RAS-G12V or FAM83B expressing HME1 cells. Therefore, FAM83B-mediated transformation could not be recapitulated by any single RAS effector.

During our original characterization of FAM83B function, we focused on a phospholipase D-like motif in the N-terminal Domain of Unknown Function 1669 (DUF1669), as it was the only clear functional motif. Indeed, we observed FAM83B-expressing cells had elevated PLD activity; however, this was not due to FAM83B itself but the hyperactivation of PLD1 [13]. Here, we show that increased PLD activity combined with constitutive CRAF activation failed to recapitulate FAM83B-mediated transformation. Thus, while PLD activity is critical for FAM83B-mediated transformation, additional signaling pathways are also required to recapitulate the transformed phenotype.

FAM83B is a member of an 8 member protein family (FAM83) that shares the highly conserved DUF1669. In addition to our identification of FAM83B, Lee et al. identified FAM83A in a cDNA library screen for proteins that confer resistance to EGFR tyrosine kinase inhibitors. Their study identified an interaction between FAM83A and the PI3K regulatory subunit p85α to activate the PI3K signaling axis, leading to AKT phosphorylation [14]. Therefore, we examined whether activated PI3K cooperated with activated MAPK signaling to recapitulate the FAM83B-transformed phenotype in HME1 cells. Indeed, constitutive PI3K/MEK activation yielded the same level of transformation as FAM83B, suggesting that FAM83B can activate both effector pathways. In addition, expression of a constitutively active PI3K or AKT with constitutively active MEK partially reversed the growth inhibition observed following FAM83B ablation. The partial rescue could result from the incomplete activity of one of the constitutively active proteins (CA-AKT, CA-PI3K, or CA-MEK) or because FAM83B activates additional, as yet undefined pathways. In any event, the finding that the FAM83 proteins activate both PI3K and MAPK signaling provides an exciting opportunity to therapeutically target both signaling pathways simply by inhibiting FAM83B. Furthermore, similar to the FAM83A studies performed by Lee et al, we identified complexes involving FAM83B:PI3K and FAM83B:AKT, enhanced membrane localization of both PI3K and AKT, and increased AKT phosphorylation. Whether the DUF1669 is responsible for complexes involving PI3K (p85α and p110α) is an interesting hypothesis since both FAM83A and FAM83B, which are quite divergent beyond the DUF1669, were able to co-precipitate with p85α. Furthermore, the conserved DUF1669 is critical for the transforming activity of FAM83B and we postulate that other FAM83 members may also function as oncogene given the conservation of this domain.

The increased PI3K/AKT activity observed in FAM83B-expressing cells, together with the activation of MAPK signaling indicates that FAM83B is an important protein in RAS effector activation (Fig. 6F). Additional significance for this observation is provided by our findings that the growth and tumorigenicity of transformed cells harboring mutant RAS or elevated EGFR expression can be significantly suppressed following FAM83B ablation. Importantly, the growth suppression engaged following FAM83B ablation is coupled with decreased CRAF, PI3K, and AKT membrane localization, and a subsequent suppression of activating phosphorylations on ERK1/2 and AKT. These results provide compelling evidence that therapeutically targeting FAM83B may inhibit both MAPK and AKT signaling simultaneously in tumors dependent on EGFR/RAS signaling. Combined therapies targeting MAPK and AKT/mTOR signaling are currently being evaluated in clinical trials for several tumor types [9]. However, the complexity of RTK and downstream signaling interactions continues to limit the effectiveness of targeted therapies aimed at inhibiting HER2, EGFR, RAS, RAF and MEK [7;8]. Previously, we demonstrated that cells expressing elevated FAM83B levels had a decreased sensitivity to EGFR-TKIs [13]. Given the requirement for FAM83B as an activator of PI3K/AKT and MAPK in EGFR/RAS signaling, the levels of FAM83B may be an important factor to consider when determining which patients receives TKI treatment [15]. Our significant observation that inhibition of FAM83B expression can suppress the activity of both CRAF and PI3K signaling provides an exciting new target for future therapies aimed at inhibiting both pathways simultaneously to inhibit tumor growth.

## MATERIALS AND METHODS

### Materials

The subcellular protein fractionation kit was purchased from Thermo Scientific and the standard protocol was used. RAF Kinase Inhibitor I (GW5074), U0126, LY294002, and Rapamycin were purchased from Calbiochem, and MK2206 was purchased from Selleck Bio., and Matrigel was purchased from BD Biosciences.

### Retroviral Constructs

pBabe-puro-cRAF-22W was kindly provided by Channing J. Der (University of North Carolina at Chapel Hill, Addgene Plasmid #12593). pcDNA5/TO-myc-PLD1 and pcDNA5/TO-myc-PLD2 were provided by H. Alex Brown (Vanderbilt University; [16]) and was subcloned into pLNCX2 (Clontech). phCMV2-HA-PLD1 and phCMV2-HA-PLD1-K830R were kindly provided by Julian Gomez-Cambronero (Wright State University) and subsequently subcloned into pLNCX2. LPCX-FLAG-FAM83B, pLKO.1-shFAM83B (#2), and pLKO.1-shGFP were previously described [13]. pBabepuro-RasV12, pBabepuro-RasV12-Y40C, pBabepuro-RasV12-T35S, and pBabepuro-RasV12-E37G (Addgene plasmids 1768, 12276, 12274, and 12275) were obtained from Addgene, deposited by Dr. Robert Weinberg (Whitehead Institute, Cambridge, MA). pBabe-puro-MEK-DD (Addgene plasmid 15268 ) was obtained from Addgene, deposited by Dr. William Hahn (Dana-Farber Cancer Institute, Boston, MA). pwzl-neo-Myr FLAG AKT1 (Addgene plasmid 20422) was obtained from Addgene, deposited by Dr. Jean Zhao (Dana-Farber Cancer Institute, Boston, MA). pwzl-puro-Myr-p110a was previously described [17]. pSV-HA-p85 (Addgene plasmid 11499) was obtained from Addgene deposited by Dr. Ronald Kahn (Harvard Medical School, Boston, MA).

### Virus Production and Infection

Retroviruses were produced as described [18;19]. Briefly, retroviral vectors were transfected into Phoenix-Ampho cells together with a packaging plasmid encoding the MLV-gag-pol and env genes. Plasmids encoding shRNAs targeting FAM83B or GFP in pLKO.1 were acquired from Open Biosystems and Sigma-Aldrich. Viruses were packaged in 293T cells using the second-generation packaging constructs pCMV-dR8.74 and pMD2G, were kind gifts from Didier Trono (University of Geneva, Switzerland). Supernatants containing virus, were collected at 24 and 48 hours and supplemented with 4 μg/ml of polybrene before being frozen in aliquots or used to infect cells for 6-24 hours.

### Cell Lines and Culture Conditions

hTERT-HME1 cells were purchased from (Clontech) and grown in a humidified atmosphere containing 5% CO_2_ at 37°C in Medium 171 with mammary epithelial growth supplement (Cascade Biologics) and 50 units/mL of penicillin and 50 μg/mL of streptomycin sulfate (U.S. Biochemical Corp.) as described [20]. HCC1937, MDA468, and HCT116 cancer cell lines were grown in a humidified atmosphere containing 5% CO_2_ in DMEM (with glucose and L-glutamine; Life Technologies) with 5% fetal bovine serum and 50 units/mL of penicillin and 50 μg/mL of streptomycin sulfate (U.S. Biochemical Corp.).

### Immunoprecipitation and Western Analysis

Immunoprecipitation experiments were carried out as previously described with exceptions as noted [20;21]. FAM83B and p85, p110, or AKT were immunoprecipitated using anti-FLAG (M2) affinity gel (Sigma Aldrich), from 293T cells transfected with 4μg of each plasmid using lipofectamine 2000 (Invitrogen) as indicated in each experiment. Whole cell extracts were prepared by incubating cell pellets in lysis buffer containing 50 mmol/L of Tris (pH 8.0), 150 mmol/L of NaCl, 1.0% NP40, 10 μg/mL of aprotinin, 100 μg/mL of phenylmethane sulfonyl fluoride, 5 μg/mL of leupeptin, 5 μg/mL of pepstatin, and 1 mmol/L of NaVO_4_. Cell extracts containing equal quantities of proteins, determined by the Bradford method, were separated by SDS-PAGE (8–12.5% acrylamide) and transferred to polyvinylidene difluoride membranes (Millipore). Antibodies to HA (F-7), MYC (9E10), HRAS(C-20), and PC-PLD1 (F-12) were from Santa Cruz Biotechnology, antibodies to ß-actin (pan Ab-5) were from Neomarkers, antibodies to glyceraldehyde-3-phosphate dehydrogenase were from Calbiochem, antibodies to FLAG (M2) were from Sigma-Aldrich and antibodies for ERK1/2, P-ERK1/2 (Thr202/Tyr204), AKT, P-AKT (Ser473), CRAF, STAT3, EGFR, PI3K, p85, and MEK1/2 were from Cell Signaling. Monoclonal FAM83B antibodies (clones 7D11 and 12G11) were generated against recombinant FAM83B protein (Hybridoma Core Facility, Cleveland Clinic). Primary antibodies were detected with goat anti-mouse or goat anti-rabbit conjugated to horseradish peroxidase (Hoffman-La Roche), using enhanced chemiluminescence (Perkin-Elmer).

### Soft Agar Assays

For hTERT-HME1 cells, 1 × 10^5^ cells were suspended in 0.6% type VII agarose (Sigma) and plated onto a bottom layer of 1.2% agar in a 60mm plate in triplicate as described [17]. The medium was changed every 3 days until cells were analyzed after 3 weeks. To quantify colonies, each plate was scanned using an automated multi-panel scanning microscope, and the digital images were analyzed using MetaMorph image quantification software. For HCC1937 cells, 2 × 10^5^ cells were plated per 60mm dish and medium was changed every 3 days until cells were analyzed after 3 weeks. The specific pharmacological PI3K inhibitor LY294002 was obtained from Sigma Aldrich and the AKT inhibitor MK2206 was obtained from Selleck Chemicals. Both compounds were added to the medium during feedings for indicated experiments.

### Relative Growth Assay

hTERT-HME1, HCC1937, and MDA468 cells were plated in triplicate at 20,000 cells/well in triplicate and cell number was determined on a Beckman Coulter counter. The RAF inhibitor GW5074, mTOR inhibitor Rapamyicn, MEK1/2 inhibitor U0126, and PI3K inhibitor LY294002 were obtained from Sigma-Aldrich; the AKT inhibitor was obtained from Selleck Chemicals and added to the indicated experiments.

### Laminin-Rich Basement Membrane (lrBM) Cultures

Growth factor reduced lrBM was purchased from BD Biosciences. LrBM cultures were performed by overlaying 120 μl of growth factor reduced lrBM onto 6 well chamber dishes and allowing it to solidify for 30 minutes at 37ºC in a cell culture incubator. Approximately 1×10^5^ cells are embedded in 880 μl lrBM and allowed to solidify for 1 hour. The solidified lrBM cultures are reefed with 1mL of complete growth media, with refeedings every 4 days for 12 days. Phase contrast images were taken at day 12 and total acini were quantified using an automated multi-panel scanning microscope, and the digital images were analyzed using MetaMorph image quantification software.

### Subcutaneous Tumorigenicity Assays

NOD/SCID mice were bred and maintained under defined conditions at the Athymic Animal and Xenograft Core facility at the Case Western Reserve University, Case Comprehensive Cancer Center an accredited facility that acts in compliance with NIH guidelines and provides veterinary care by several full time veterinary personnel. HCT116 (1×10^6^) cancer cells were suspended in a 1:1 mixture of media and matrigel (BD Biosciences) and injected subcutaneously into mice (6-8 weeks of age) that were sublethally irradiated with 300 rad 4 hours prior to injection. Tumor volume was calculated with the formula 4/3πr3 and student t-tests were performed.

## Supplementary Tables


