# Supplementary material for: FAM83B-mediated activation of PI3K/AKT and MAPK signaling cooperates to promote epithelial cell transformation and resistance to targeted therapies
Source: Oncotarget. 2013 May 11;4(5):729–38. doi: 10.18632/oncotarget.1027 (PMC3742833; doi:10.18632/oncotarget.1027)
Supplement: Supplementary file 1 [file oncotarget-04-729-s001.pdf]

Supplementary Figures

FAM83B-mediated activation of PI3K/AKT and MAPK signaling cooperates to promote epithelial cell transformation and resistance to targeted therapies.

Rocky Cipriano, Kristy L.S. Miskimen, Benjamin L. Bryson, Chase R. Foy, and Mark W. Jackson

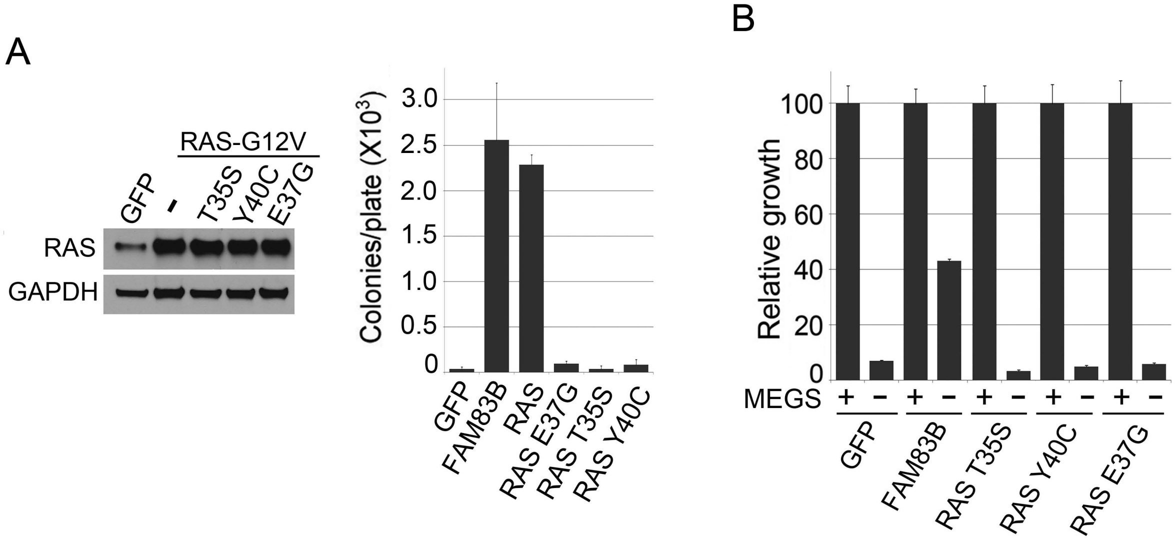

Supplementary Figure 1. FAM83B utilizes multiple RAS effectors to induce AIG. (a) HME1 cells expressing GFP, FAM83B, RAS-V12, RAS-V12-E37G (RAL-GEF), RAS-V12-T35S (RAF), or RAS-V12-Y40C (PI3K) were plated in soft agar to assess AIG and western analysis was performed to confirm expression. (b) HME1 cells expressing GFP, FAM83B, or RAS-V12 point mutants were plated in the presence and absence of mammary epithelial growth supplement (MEGS) and cell number quantified 5 days later.

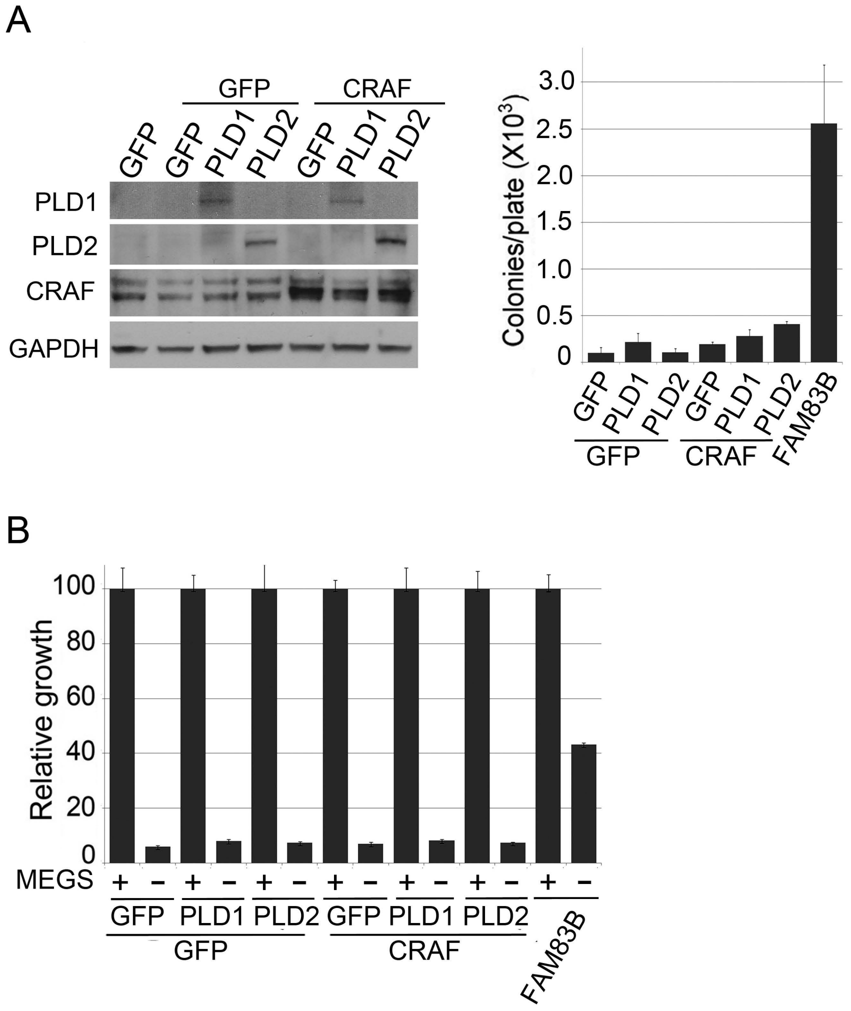

Supplementary Figure 2. Constitutively active CRAF and elevated PLD activity are insufficient to recapitulate FAM83B phenotypes. (A) HME1 cells expressing GFP or constitutively active CRAF were infected with retroviruses encoding cDNAs of GFP, PLD1, or PLD2 and western analysis and AIG was assessed. HME1 cells expressing FAM83B were used as a positive control. (B) HME1 cells expressing GFP or constitutively active CRAF were infected with retroviruses encoding cDNAs of GFP, PLD1, or PLD2 and were plated in the presence and absence of mammary epithelial growth supplement (MEGS) and cell number quantified 5 days later. HME1 cells expressing FAM83B were used as a positive control.
